# Prediction of Human Brucellosis in China Based on Temperature and NDVI

**DOI:** 10.3390/ijerph16214289

**Published:** 2019-11-05

**Authors:** Yongqing Zhao, Rendong Li, Juan Qiu, Xiangdong Sun, Lu Gao, Mingquan Wu

**Affiliations:** 1 Institute of Geodesy and Geophysics, Chinese Academy of Sciences, Wuhan 430077, China; zhaoyongqing@asch.whigg.ac.cn (Y.Z.); qiujuan@asch.whigg.ac.cn (J.Q.); 2 University of Chinese Academy of Sciences, Beijing 100049, China; 3 Department of geological mapping engineering, Shanxi Institute of Energy, Yuci 030600, China; 4 China Animal Health and Epidemiology Center, Qingdao 266032, China; gaolu@cahec.cn; 5 The State Key Laboratory of Remote Sensing Science Institute of Remote Sensing and Digital Earth, Chinese Academy of Sciences, Beijing 100101, China; wumq@aircas.ac.cn

**Keywords:** brucellosis, remote sensing, time-series, SARIMAX

## Abstract

Brucellosis occurs periodically and causes great economic and health burdens. Brucellosis prediction plays an important role in its prevention and treatment. This paper establishes relationships between human brucellosis (HB) and land surface temperature (LST), and the normalized difference vegetation index (NDVI). A seasonal autoregressive integrated moving average with exogenous variables (SARIMAX) model is constructed to predict trends in brucellosis rates. The fitted results (Akaike Information Criterion (AIC) = 807.58, Schwarz Bayes Criterion (SBC) = 819.28) showed obvious periodicity and a rate of increase of 138.68% from January 2011 to May 2016. We found a significant effect between HB and NDVI. At the same time, the prediction part showed that the highest monthly incidence per year has a decreasing trend after 2015. This may be because of the brucellosis prevention and control measures taken by the Chinese Government. The proposed model allows the early detection of brucellosis outbreaks, allowing more effective prevention and control.

## 1. Introduction

Brucellosis is a common zoonosis afflicting humans and animals and causes heavy economic and health burdens around the world [1,2,3]. There are four types of brucellosis [4,5] affecting sheep, cows, pigs, and dogs, with transmission to humans mainly occurring through close contact with infected animals and their wastes, or by eating unsterilized animal products such as fresh milk [4,6,7,8]. Aborted sheep fetuses are highly infectious. Farmers, butchers, and veterinarians who come into contact with animals are at high risk of infection. Brucellosis often causes a series of symptoms in infected people [9,10], and it leads to animal production and abortion [11,12,13,14]. Brucellosis is mainly distributed in Asia, the Middle East, sub-Saharan Africa and the Balkan Peninsula [1,15,16]. More than 500,000 cases occur globally every year [17]. Sheep brucella vaccine rev.1 is not suitable for human use [18,19]. The United States, New Zealand, and other countries have adopted quarantine or culling measures [20,21] but these measures cannot eradicate brucellosis completely.

As a country with a vast agricultural industry, China’s brucellosis prevention and control situation is very serious [13]. The number of brucellosis cases is on the rise and the affected region is gradually spreading from the north to the south. The north is a high-incidence area [22], especially in Inner Mongolia. Meanwhile, the south has sporadic outbreaks [23], especially in recent years in Hainan Province, which was previously free of the disease. The first case of brucellosis occurred in China in 1905 and high rates of incidence occurred before 1980 that have since improved [13,24]. This is related to a series of measures taken by the Chinese Government: (1) A reporting system was established in 1950 [13]; (2) vaccination began in 1964 in high-risk areas; (3) a definition of the disease, test standards, control measures, and treatment programs were formulated in 1977; and (4) in 1980, a monitoring project of serum-positive rates at brucellosis sentinel sites was implemented. However, from the middle to late 1990s to the beginning of this century, the incidence of brucellosis increased sharply and has continued to increase in recent years, which presents great challenges to public health and social economic systems. Therefore, new theories and forecasting methods are urgently needed to meet these challenges.

Previous publications have focused on the epidemiological characteristics and spatial distribution of the disease [15,25,26,27], while time series-based predictive models have been used to provide early warnings of disease occurrence. Common models include residual autoregression models, the exponential smoothing method (ES) [28], grayscale models, negative binomial regression models, artificial neural network models [26], autoregressive integrated moving average models (ARIMAs) [29,30], and their syntheses [31]. ARIMA models are optimal when the time series contains long-term trend, periodicity, and disturbance terms [32,33,34,35]. Previous ARIMA models only considered the model itself without considering the influence of other variables. An improved ARIMA model, the autoregressive integrated moving average with exogenous variables (ARIMAX) model, overcomes this defect by incorporating other factors. Some epidemics involve factors such as hydatids [36] and *Oncomelania* snails. These factors have been modelled in other papers using factors such as temperature, precipitation, insolation time, relative humidity, average wind speed, vegetation cover, etc. [24]. Remote sensing has been applied in many fields as it can estimate many of these factors [16,37,38,39,40,41,42,43]. Although ARIMAX has been used to study air pollution [44], corn yields [45], and to predict various illnesses [46], no one has used it to study brucellosis in recent years. In addition, the use of ARIMAX models helps to explore the relationship between time series and other factors. Therefore, this study aims to fill this research gap. Brucellosis bacteria are particularly sensitive to temperature [26], especially surface temperature. In addition, vegetation cover can provide suitable hydrothermal conditions for bacterial survival. In this paper, on the basis of predecessors’ ARIMA models that consider seasonal factors, we use remote sensing data on land surface temperature and vegetation cover to construct an ARIMAX model of the short-term onset of brucellosis.

The aims of this paper are to: (1) Establish an ARIMAX model using surface temperature and vegetation cover as input variables; and (2) use it to predict the incidence of brucellosis in mainland China.

## 2. Materials and Methods

### 2.1. Data Sources

#### 2.1.1. Human Brucellosis Data

Data on human brucellosis (HB) cases were collected on a monthly basis by the Chinese Centre for Disease Control and Prevention (CDC) [47] from January 2011 to December 2016. Brucellosis is a class b infectious disease, as stipulated by the law of prevention and treatment of infectious diseases in China. Cases of brucellosis must be reported within 24 h and suspected cases are subject to clinical and serological testing or isolation as required by the World Health Organization. In this study, monthly means of HB cases across China from January 2011 to May 2016 were used in the model to fit the ARIMAX model, while the data from June 2016 to May 2017 were used for validation.

#### 2.1.2. Land Surface Temperature Data

Land surface temperature (LST) has an important impact on surface-dwelling organisms and is an important variable for studying biological change. In the present study, LST data were obtained for January 2011 to May 2016, and monthly means were used as input for the ARIMAX model of brucellosis. The data was provided by the International Scientific Data Mirror website [48] of the Computer Network Information Centre of the Chinese Academy of Sciences. We used the MODLT1T China 1 km surface temperature monthly composite product. Then, ArcGIS10.4 software (Environment Systems Research Institute, Redlands, CA, USA) was used to obtain monthly mean daytime and night-time surface temperatures across China (the monthly product includes daily and evening monthly average images), and a monthly mean national temperature was calculated.

#### 2.1.3. Normalized Difference Vegetation Index Data

The normalized difference vegetation index (NDVI) is an important indicator of land cover. It is provided by the International Scientific Data Mirror website [48] of the Computer Network Information Centre of the Chinese Academy of Sciences. The MODND1M China 500 m NDVI monthly synthesis product from January 2011 to May 2016 was adopted, and ArcGIS software was used to calculate national mean monthly NDVI values.

### 2.2. Methods

#### 2.2.1. General Concepts

**Figure 1:**
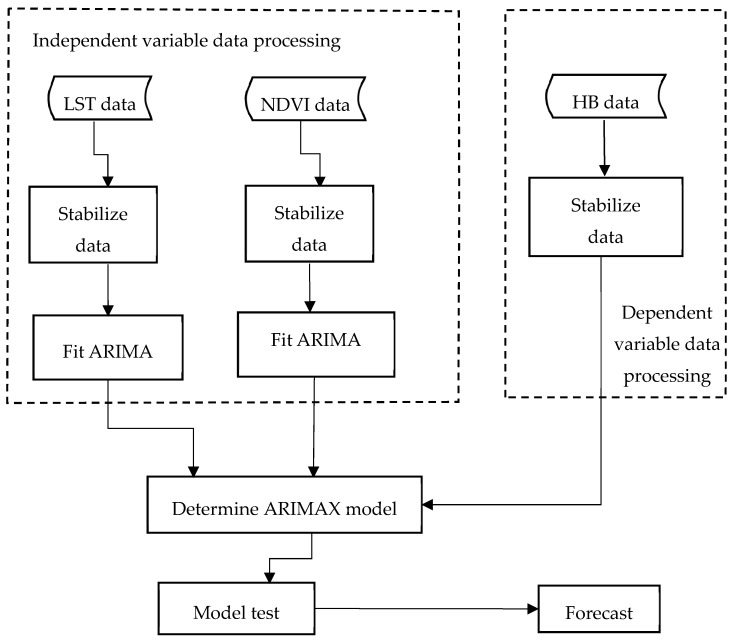
Data processing and modelling flowchart. LST represents land surface temperature, NDVI expresses normalized difference vegetation index, HB indicates human brucellosis.

The steps used to build the overall model are as follows. The input HB, LST and NDVI data are differentiated to stabilize them. Independent variables of LST and NDVI are fitted to the ARIMA model. Each independent variable model is used to filter the HB data and determine a fitted model, as shown in Figure 1. The detailed data processing procedure is shown in the following section.

#### 2.2.2. Seasonal Autoregressive Integrated Moving Average with Exogenous Variables (SARIMAX) Models

As a classical time series model [29], ARIMA (p, d, q) is a further development of the AR (p) and MA (q) models, where p is the number of AR autoregression terms, d is the difference order, and q is the number of MA sliding average terms. If the information of the sequence cannot be fully extracted when the time series is stable, an $SARIMA(p,d,q){\left(P,D,Q\right)}_{S}$ model [49] needs to be considered, where *P* refers to the maximum lag order of the seasonal autoregression term, *Q* is the maximum lag order of the moving average operator, *D* is the seasonal difference order, and *S* is the seasonal difference cycle step. When we consider input variables such as LST, NDVI, we need to use an ARIMA model with input variables, namely, an ARIMAX model. A SARIMAX model can also be considered to be an ARIMA model with an interference sequence or dynamic regression model, which contains a regression section with input variables and an ARIMA section. Its general model is shown as formula (1):

(1) $$\{\begin{array}{c} y_{t}=\mu +\sum_{i=1}^{k}\beta_{i}B^{li}x_{it}+\varepsilon_{t}\\ \varepsilon_{t}=\frac{\theta_{q}(B)\Theta_{Q}(B^{S})\;}{\phi_{p}(B)\Phi_{P}(B^{S}){\left(1-B\right)}^{d}{\left(1-B^{S}\right)}^{D}}\;a_{t} \end{array}$$

Here, μ represents a constant, $\beta_{i}$ is regression coefficients of external variables, $\Phi_{P}\left(B\right)$ refers to the seasonal autoregressive coefficient polynomial with P-order, $\Theta_{Q}\left(B\right)$ denotes the polynomial of the seasonal moving average coefficient with Q-order, $\phi_{p}(B)$ stands for the autoregressive coefficient polynomial with p-order, $\theta_{q}(B)$ is the polynomial of the seasonal moving average coefficient with q-order, ${\left(1-B\right)}^{d}$ indicates differencing operator of order *d*, ${\left(1-B^{S}\right)}^{D}$ remarks seasonal differencing operator of order D, S expresses the seasonal length, $\varepsilon_{t}$ shows the residuals, $a_{t}$ is white noise time series, $\mu +\sum_{i=1}^{k}\beta_{i}B^{li}x_{it}$ suggests the regression part, and $\varepsilon_{t}$ indicates the disturbance part.

**Figure 2:**
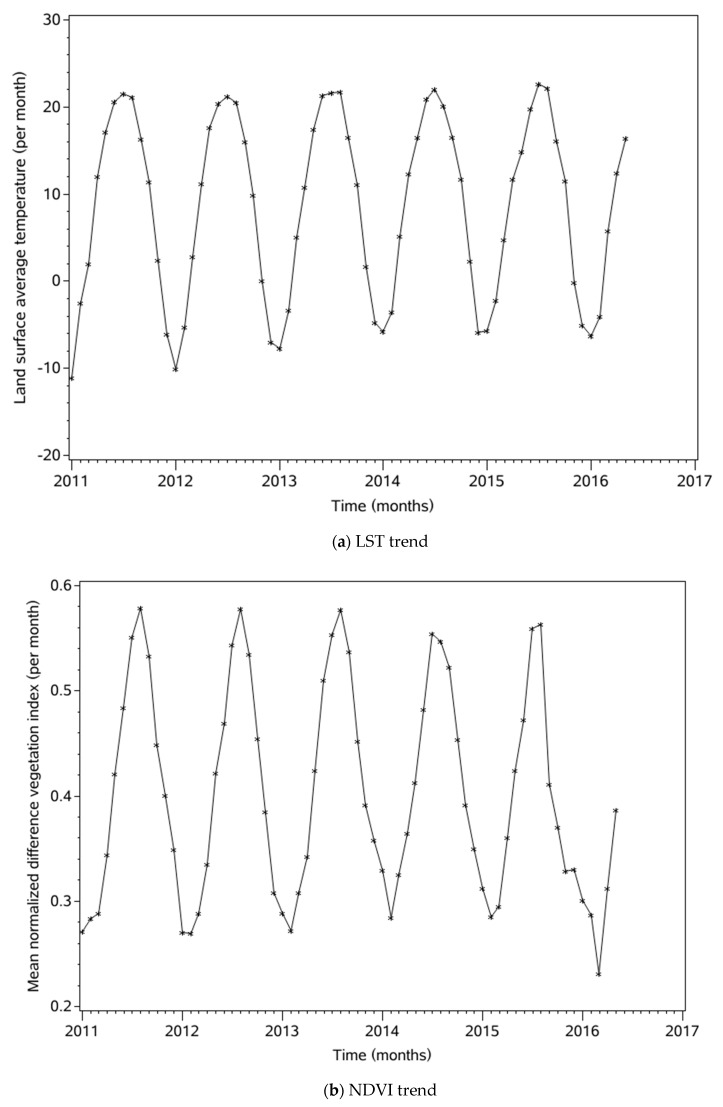
Trends of input variables, (**a**) LST trend, (**b**) NDVI trend.

When setting up an ARIMAX model, the steps are as follows: (1) A stationarity test is carried out for the response sequence and each input variable. If the input data is not stationary, it should be stabilized by a differential method. No matter which difference [50] method is adopted, it cannot meet the requirement of stationarity and the seasonal variation is obvious. Therefore, a seasonal difference can be considered. Using a seasonal differential method, the sequence was determined to be in a steady state. Since the surface temperature data and NDVI (Figure 2) are themselves in the stationary condition, the next step can be taken directly. Similarly, HB data requires both first-order difference and seasonal difference processing to be a stationary series. The method used to check whether the data is stationary is the Augmented Dickey-Fuller (ADF) test [51], with a result of *p* < 0.05 being indicative of stability. (2) White noise testing. A stationary time series is of no value if it is simply white noise. On the condition that it is not white noise, further study is necessary, then further pattern recognition. All the above three input variables are non-white-noise sequences. (3) ARIMA and prewhitening. The ARIMA model is fitted to each input variable—LST, NDVI—to make the residual of the model a white noise sequence. Then, the output variable brucellosis sequence is filtered by this model to calculate the correlation coefficient between the input sequence and the output sequence. (4) The structure of the SARIMAX model is determined by using the co-correlativity coefficient, and then the model is fitted. (5) Checking the fitted model. If the requirements are not met, adjustment continues until the white noise test and the significance of the parameters meet the required conditions.

#### 2.2.3. Model Test

The root mean square error (RMSE), mean absolute percentage error (MAPE), and mean error rate (MER) are used to evaluate the fit and predictive effect of the established model. These are calculated according to Equations (2)–(4).

(2) $$\mathrm{RMSE}=\sqrt{\frac{1}{N}\sum_{i=1}^{N}{\left(X_{i}-{\overset{}{\overset{⌢}{X}}}_{i}\right)}^{2}}$$

(3) $$MAPE=\frac{1}{N}\sum_{i=1}^{n}\frac{\left|X_{i}-\overset{⌢}{X_{i}}\right|}{X_{i}}$$

(4) $$MER=\frac{\frac{1}{N}\sum_{i=1}^{N}\left|X_{i}-{\overset{⌢}{X}}_{i}\right|}{{\bar{X}}_{i}}$$

In the formulas, $X_{i}$ denotes actual values, ${\bar{X}}_{i}$ is the mean of the actual values, ${\overset{⌢}{X}}_{i}$ is the fitted or predicted values, and *N* refers to the number of simulated or predicted values. The software used in this study was SAS 9.4 (SAS Institute Inc., Cary, NC, USA), ARCGIS 10.4 and EXCEL 2016 (Software, Redmond, CA, USA). The LST and NDVI data were read by ARCGIS 10.4, and the BDI data were imported into EXCEL 2016. Data fitting, model establishment, inspection, and mapping were all carried out in SAS 9.4.

## 3. Results

### 3.1. General Characteristics

**Figure 3:**
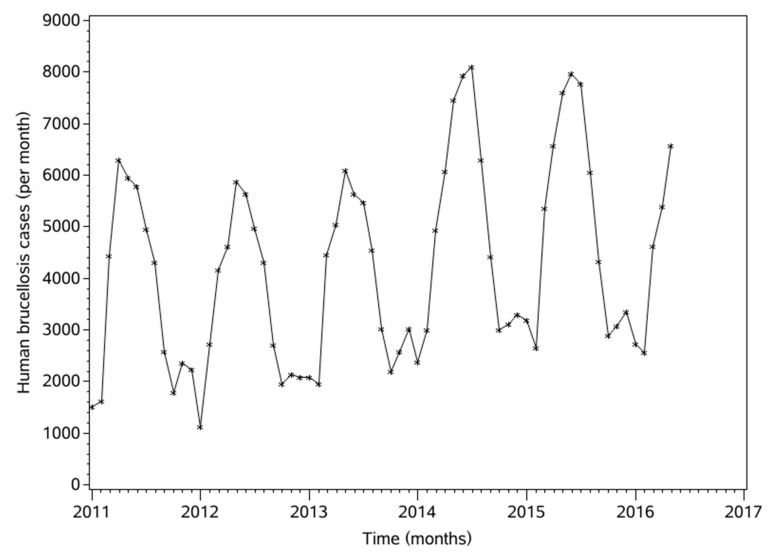
Trends in monthly human brucellosis cases.

A total of 71 observed values of brucellosis data were obtained from January 2011 to November 2016, among which the first 65 were used as training data for the establishment of an ARIMAX model, and the last six values were used for comparison to test the predictive effect of the model. Looking at the data from previous years, the monthly data ranged from a minimum of 1123 cases per month (January 2012) to a maximum of 8102 cases per month (July 2014). The total number of cases in more than five years was 274,800, the monthly average was 4227 cases per month (HB/mo), and the standard error was 1875 HB/mo. Between 2011 and 2015, HB cases increased from 43,827 to 60,782, an increase of 138.68%. According to the data, the incidence of brucellosis has obvious seasonality and periodicity, with a peak period of May to August, as shown in Figure 3.

### 3.2. HB Data Stationarity

As a response sequence, HB data is required to be stationary for modelling purposes. It can be seen from the scatter diagram that it is non-stationary and has obvious seasonality. After the difference method is adopted, it is found that the data is first order twelve-step differential stability. The test results are shown in Table 1.

### 3.3. LST and NDVI Models

A scatter diagram of surface temperature and NDVI data from January 2011 to May 2016 was made and it showed that the data were approximately stable. The ADF test result of *p* < 0.05 showed that the data were stable, so the difference method was not needed. Meanwhile, the white noise test showed a non-white noise sequence. According to the ACF diagram, PACF diagram, and AIC criterion, the optimal temperature model is ARMA (2, 1), whose corresponding AIC and SBC values are 340.43 and 349.13, respectively. All parameters of this model are significant, and the residual error is white noise. The NDVI ADF test shows that the NDVI model is stable and the residuals are white noise. Also, with the aid of the ACF and PACF diagrams, the orders of models were determined. Finally, the selected models are shown in Table 2; the optimization model is ARMA (2, 1). The results for LST and NDVI are shown in Figure 2.

### 3.4. SARIMAX Model

**Figure 4:**
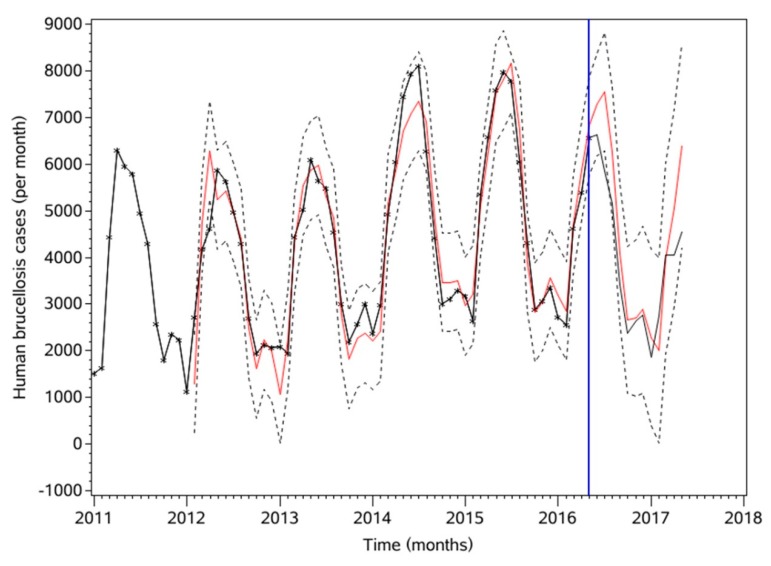
Brucellosis fitting and prediction (January 2011–May 2017). The black line represents actual observations. The red line to the left of the blue vertical line represents fitted values, while that to the right of the blue line represents predicted values. The dotted black lines represent 95% confidence intervals.

The optimal brucellosis model was filtered by the optimal models of LST and NDVI, then a regression equation was determined, with the model fitted according to the correlation number. If the parameters of the fitted model are not significant or the residuals do not pass the white noise test, the parameters need to be adjusted constantly. Finally, the model with significant parameters (Table 3) that passed the white noise test (Table 4) after multiple adjustments is shown as Formula (5), and its corresponding AIC and SBC values are 807.58 and 819.28, respectively. This model is used for prediction, with the predicted results shown in Table 5 and in Figure 4. After establishing the final model, RMSE, MAPE and MER are adopted to evaluate the ARIMAX model established above.

(5) $$(1-B)(1-B^{12})y=(-36.80113\begin{array}{r} +\begin{array}{l} 25.29822 \end{array} \end{array}B)x_{1}+(\begin{array}{l} 5000.2 \end{array}-4680.1B)x_{2}+(1-0.53724B)(1-0.57166B^{12})\varepsilon_{0}$$

In the formula, *y* represents the incidence of HB, *x*_1_, *x*_2_ represent the input variables LST, NDVI, respectively, *B* is a lag factor, and $\varepsilon_{0}$ is the white noise residual sequence.

### 3.5. Assessing Model Performance

For the final evaluation result, RMSE, MAPE, MER [29], and other common evaluation methods are adopted to evaluate the difference between fitted and predicted values. For the fitted values, the RMSE = 506.896, MAPE = 0.104, and MER = 0.089. For the predicted values, RMSE = 921.215, MAPE = 0.179, and MER = 0.188. The predicted results are shown in Table 5.

## 4. Discussion

### 4.1. Trends and Seasonal Periodicity

As can be seen from Figure 1, the annual incidence of brucellosis has been increasing since 2011, with the annual peaks increasing. One important cause is the increasing scale of livestock husbandry caused by improvements in China’s economy and demands for livestock products in recent years [52]. At the same time, it may be related to the free-range breeding methods used by farmers, which have much higher animal disease risks than conventional methods. In this case, susceptible animals can come into contact with infected animals or brucellosis bacteria. Sheep are the main source of infection in China, followed by cattle and pigs. The peak timing of brucellosis rates is also closely related to the lamb production season. The LST, NDVI, and HB data have similar trends, which indicates that there is a close relationship between them. See Figure 3 for more details.

Brucellosis shows obvious seasonal periodicity. The incidence of brucellosis is relatively high from April to August every year, with the peak occurring from May to July. This may be related to many factors [36], such as precipitation, lamb production, animal movement, wind speed, and sunshine levels. A more plausible explanation is that as temperatures rise in the spring, snow and ice melt, amounts of organic matter increase, and precipitation increases due to weather changes, all of which are conducive to the activation and incubation of bacteria. Spring is also the breeding season for many animals. With more animals moving around, the area affected by infected animals and their excretions becomes larger and the risk of infection of sensitive animals is higher. The net result may be a growing number of brucellosis cases that are distributed more widely across the country.

It can be seen from Figure 3 that from January 2011 to May 2016, parts of the fitted results are almost identical to the actual values. Only in July 2014 do the fitted and actual values have certain differences; the month where the abnormal values reached 8102, and which attained a maximum from 2011 to 2016. This is because the results of the ARIMAX simulation are continuous and smooth, so extreme values or outliers will make it relatively flat. From January 2011 to May 2016, the annual maximum values showed an increasing trend, however, the ARIMAX model could accurately predict from this increasing trend that the maximum monthly incidence decreased in 2016. It was less than in 2014 and 2015. This is consistent with actual observations, indicating that this model predicts trends well. In addition, since 2015, the rate of brucellosis cases has shown a downward trend. One important reason is that the measures taken by the Chinese Government to control brucellosis more strictly have been effective.

### 4.2. The Interrelations of Variables and Their Significance

Since LST and NDVI are two environmental factors that have important influence on HB, we chose these two factors mainly to explore their influence on HB. Although collinearity exists between LST and NDVI, collinearity does not affect the prediction accuracy of ARIMAX model. So, we do not deal with it. As shown in Figure 4, the fitted curve is basically consistent with that of NDVI. The coefficient of NDVI is the largest, indicating that its change does indeed indicate the basic trend of brucellosis onset and has the greatest influence. This is because vegetation coverage increases from January to July each year. This provides live *Brucella* bacteria with favorable hydrothermal equilibrium conditions. With increasing areas of favorable conditions, the number of *Brucella* hosts, such as sheep that feed on infected vegetation, increases. The highest incidence of brucellosis occurs in Inner Mongolia [23] in northern China, which is closely related to its widespread grasslands. This is the best example of the influence of vegetation on *Brucella*. Because the time at which spring ewes lamb is appropriate for *Brucella*, contact with amniotic fluid that carries *Brucella* bacteria can cause infection in humans. However, with increases in temperature, water evaporation increases and large numbers of bacteria multiply rapidly. The moisture content of the soil decreases because of water evaporation. At the same time, brucella has very high nutritional demands, while a large number of bacteria are competing for nutrients from brucellosis bacteria, so *Brucella* bacteria receive less nutrition. These conditions do not facilitate the growth of bacteria, causing brucellosis cases to decrease. Hence, there is a negative correlation between LST and brucellosis cases. In summary, HB incidence is primarily determined by NDVI, while LST influence the amplitude of its change. Previous studies have considered temperature, moisture, and other environments in which bacteria infect hosts; however, it is better to also consider the effect of vegetation on temperature and moisture.

High vegetation coverage is good for *Brucella* survival and spread, which is a kind of average result. Some high vegetation coverage and wide distribution is very good for *Brucella* bacteria survival, while some other coverages are not very good. The level of NDVI that causes the highest HB incidence requires further study. The Inner Mongolia prairie [23] is very favorable for *Brucella* survival and the highest HB incidence in China is found there. In some southern areas, the vegetation coverage is higher but is not necessarily suitable for *Brucella* survival. From the local area to the national level, no vegetation types were significantly adverse to brucellosis. Of course, some vegetation may play a greater role in *Brucella* survival, while others may play a smaller role.

The results of this study improve on previous research results [29]. NDVI, LST were found to be of certain significance in the prediction, prevention and control of brucellosis. The consistency of the NDVI and brucellosis incidence trends show that NDVI is of important significance in formulating accurate prevention and control strategies, as it can be used to predict the incidence of brucellosis in a country or a region according to its specific vegetation. The lagged effects of LST show that we need to allocate medical resources, personnel, and animal control and isolation measures according to changes in LST. According to these conditions, brucellosis prevention and control measures can be planned in advance, medical resources can be allocated appropriately, and public awareness can be carried out. The two variables above may have greater significance for certain regional infections due to their relatively easy availability, extensive coverage and close correlations with various surface-dwelling bacteria.

This model is established throughout the whole country and has certain applicability to high-risk areas. For high-risk areas, the idea and method of building the model are the same, but the coefficients of the model may be different. As long as we get LST and NDVI for these regions, we can build a similar model. In areas with high vegetation coverage (such as grasslands) and dense flocks of sheep, the incidence is high, such as in the grasslands of Inner Mongolia (as can be seen from Equation (5)), which is a typical high-risk area. A higher NDVI coefficient may be obtained from the model if these regions are selected, because the temperature in the north is lower and the influence of LST is greater. That is, the absolute value of the coefficient is smaller (because it is negative) and lower temperatures are more conducive to the survival of brucellosis. However, in some areas in the south, the number of sheep is relatively small and the area of vegetation cover (such as grassland) is smaller, so the incidence in these areas is relatively low. The NDVI coefficient of the model presented here is relatively small. Meanwhile, due to the high temperature in the south, LST will increase while its influence on the equation will decrease. In other words, its coefficient will decrease (the absolute value will increase).

### 4.3. Other Factors

In the analysis above, only significant factors were selected for research. Choosing the main influencing factors can help to grasp the essence of the problem and highlight its regularity so as to solve it. In addition to the above factors, there may be other factors that affect brucellosis incidence, such as relevant policies and animal numbers. In general, strict control policies may decrease the incidence of brucellosis while lax ones may increase it. From the 1970s, the incidence of brucellosis declined steadily; however, after the 1990s, the number of brucellosis cases increased continuously. After 2015, brucellosis began to decline again. These changes were related to the policies of the Chinese Government. Before the 1990s, China’s brucellosis control was strict and brucellosis incidence steadily declined. After the 1990s, due to the relaxation of awareness, vigilance and policies related to brucellosis, the number of brucellosis cases increased, and the Government attached greater importance to it. After 2013, the Government gradually formulated and adopted stronger policies that had an effect, as suggested by the decline in brucellosis since 2015.

From the perspective of animal numbers, after the 1990s, the number of livestock increased significantly. This was coupled with a restructuring of the livestock industry; the proportion of pork production decreased, while that of beef and mutton increased. According to official annual data released by the National Bureau of Statistics, the numbers of sheep raised from 2011 to 2015 were 28,664.15 (×10,000 head), 28,512.74, 28,935.22, 30,391.28, 31,174.27, and 29,930.54, respectively; an overall increasing trend. In the same period, the numbers of cattle were 9384.00 (×10,000 head), 9137.25, 8985.76, 9007.28, 9055.79, and 8834.49, respectively; an overall decrease, and contrary to the trend in brucellosis incidence. The incidence of brucellosis in sheep greatly increased along with the large increase in sheep numbers, while the incidence of brucellosis in humans remained unchanged. At the same time, the incidence of brucellosis in sheep greatly increased due to its wide distribution range and strong pathogenicity, so the chance of contracting brucellosis in humans would also have been greatly increased. This indicates that sheep and brucellosis are closely related. In 2016, the number of sheep decreased and brucellosis incidence declined. At the same time, the timing of human brucellosis incidence coincided with the timing of sheep reproduction. From 2011 to 2016, cattle numbers showed a downward trend, which was opposite to the increase in human brucellosis, indicating that brucellosis in cattle did not affect trends in human brucellosis on the whole, indicating that the influence of cattle brucellosis on human brucellosis is relatively weak. The reason may be that cattle *Brucella* are less pathogenic and only occur locally. This suggests that the effect of sheep brucellosis on human brucellosis is much greater than that of cattle brucellosis.

On the one hand, *Brucella* is dependent on the spread of environmental factors, etc. High vegetation cover provides a suitable living environment for *Brucella*. People and animals are easily infected with *Brucella* after coming into contact with these infected substances, thus greatly increasing the risk of human infection. On the other hand, exposure is also an important factor. Farmers who keep sheep on their farms may also be at increased risk of contracting brucellosis due to frequent contact with sheep. Similarly, butchers who often come into contact with live animals such as sheep during slaughter are also susceptible to infection. Therefore, the incidence of the disease is higher in the northern areas where sheep are raised intensively. Because farm households are more exposed to animals such as sheep during lambing season, this leads to a higher incidence of brucellosis, which shows a distinct seasonality.

### 4.4. Limitations

There are some shortcomings to the present study. First, the effect of precipitation on brucellosis was not considered. We know that precipitation and temperature are two important factors affecting brucellosis disease. However, since we mainly studied the problem based on remote sensing data, we did not consider precipitation, which should be done in future. In addition, since the LST and NDVI data were only available from January 2011 to May 2016, further data accumulation may be able to further improve the models.

## 5. Conclusions

In recent years, brucellosis rates have stayed at high levels, and the disease is involved in almost all provinces of China. It is seriously dangerous to human health and causes huge economic losses to the country; therefore, its control is of great significance. This study established relationships between HB incidence and several influencing factors. Using remote sensing data, we established the specific relationships between HB and LST and NDVI using an ARIMAX model. NDVI has a positive correlation with HB and follows its basic trends, while LST are negatively correlated with HB and only affect its magnitude. Although brucellosis has been on the decline since 2015 because of the prevention and control efforts of the Chinese Government, the disease is still considered serious due to its continued high incidence. Accordingly, we propose the following suggestions: (1) In the process of risk control and prevention, vegetation coverage should be regarded as an important reference index, especially in grasslands with high vegetation coverage. (2) Due to the higher human incidence in the lambing season, management of livestock practices should be strengthened at this time to prohibit the discharge of amniotic fluid and associated pollutants. (3) Since the low temperature of LST is conducive to the survival of brucellosis, during low spring temperatures, pathogens are spread in water due to snow and ice melts, resulting in greatly increased pathogen spreading and risk. Therefore, attention should be paid to strengthening the prevention and control of brucellosis at this time. (4) Since brucellosis has appeared repeatedly in China, prevention and treatment measures should be constantly reviewed to prevent decreases in the awareness of the problem. Our study has established a specific relationship between HB and several of its influences, which is of certain significance for the prevention and control of brucellosis in China. It also provides a reference for similar research in other countries.

## Figures and Tables

**Table 1:** Augmented Dickey-Fuller Unit Root Tests.

Type	Lags	Rho	Pr < Rho	Tau	Pr < Tau	F	Pr > F
Zero Mean	0	−69.6921	<0.0001	−11.03	<0.0001		
	1	−110.947	0.0001	−7.51	<0.0001		
	2	−90.4384	<0.0001	−5.33	<0.0001		
	3	−76.5233	<0.0001	−3.75	0.0004		
Single Mean	0	−69.7764	0.0004	−10.96	0.0001	60.15	0.001
	1	−111.796	0.0001	−7.46	0.0001	27.81	0.001
	2	−90.5227	0.0004	−5.26	0.0001	13.91	0.001
	3	−77.2761	0.0004	−3.71	0.0067	6.89	0.0042
Trend	0	−70.1527	<0.0001	−10.87	<0.0001	59.35	0.001
	1	−116.393	0.0001	−7.56	<0.0001	28.57	0.001
	2	−107.518	0.0001	−5.74	0.0001	17.26	0.001
	3	−112.044	0.0001	−4.11	0.0113	8.59	0.0126

**Table 2:** LST and NDVI models.

Variable	Model	AIC Value	SBC Value	Optimization Model
LST	ARMA (2, 1)	340.43	349.13	ARMA (2, 1)
NDVI	ARMA (2, 0)	237.51	230.99	ARMA (2,1)
	ARMA (2, 1)	253.63	244.94	
	ARMA (3, 2)	249.83	236.79	

Note: AIC= Akaike Information Criterion.

**Table 3:** Parameter significance test for HB by conditional least squares estimation.

Parameter	Estimate	Standard Error	*t*-Value	Approx	Lag	Variable	Shift
				Pr > |t|			
MA1,1	0.53724	0.12945	4.15	0.0001	1	HB	0
MA2,1	0.57166	0.14307	4.00	0.0002	12	HB	0
NUM1	−36.80113	16.58224	−2.22	0.0314	0	LST	0
NUM1,1	−25.29822	12.04109	−2.10	0.0412	1	LST	0
NUM2	5000.2	1720.1	2.91	0.0056	0	NDVI	0
NUM1,1	4680.1	1571.9	2.98	0.0046	1	NDVI	0

**Table 4:** Autocorrelations with lags.

Lag	χ ^2^	*df*	Pr > χ ^2^	Autocorrelations					
6 m	1.12	4	0.8908	0.052	−0.118	0.022	0.047	−0.009	−0.020
12 m	3.26	10	0.9746	−0.036	0.063	−0.099	−0.058	0.117	−0.007
18 m	7.37	16	0.9654	−0.081	−0.015	0.038	0.153	0.144	0.019
24 m	10.26	22	0.9837	−0.079	0.002	−0.051	−0.097	−0.092	0.066

**Note: *df*** = degree of freedom.

**Table 5:** Brucellosis prediction results.

Time	Forecast	95% Confidence Limits	
June 2016	7270.13	6170.46	8372.49
July 2016	7512.87	6285.99	8820.31
August 2016	6194.6	4859.57	7645.41
September 2016	4070.9	2598.44	5576.09
October 2016	2621.38	1086.38	4242.86
November 2016	2712.06	1017.16	4374.35
December 2016	2948.77	1096.85	4673.4
January 2017	2285.18	382.38	4166.83
February 2017	1988.04	33.07	3989.59
March 2017	3884.78	1925.2	6020.5
April 2017	5013.8	2947.99	7172.93
May 2017	6347.48	4193	8560.69
